# The therapeutic landscape for gut bacterial infections: opportunities and limitations

**DOI:** 10.1080/19490976.2020.1825076

**Published:** 2020-10-23

**Authors:** Adam Weiss

Dear Reader,

We are delighted to present a Special Focus on gut bacterial infections, **Guest-Edited by Dr. V. K. Viswanathan** of University of Arizona. The three articles in this section review the progress and challenges in today’s research on antimicrobial resistance, prophylactic, and antivirulence strategies for the infections of the gut.

*Clostridioides difficile*, one of the most significant pathogens of the gastrointestinal tract, causes diarrhea and, in some instances, fatal pseudomembranous colitis. *C. difficile* infection (CDI) is often a consequence of microbiota imbalance, *e.g*., following antibiotic intake. Yet, antibiotics have long been the standard-of-care treatment of CDI leading to widespread antibiotic resistance. Stewart *et al*.^1^ review alternative approaches to CDI treatment, including probiotics, antisense oligonucleotides, passive immunization, and others.

Antimicrobial resistance (AMR) is an increasing problem not only in CDI but also in other pathogens. The World Health Organization has included it in its list of top 10 threats to global health, and deaths from AMR infections are projected to reach 10 million annually by 2050. Wallace *et al*.^2^ review AMR in enteric bacteria, including pathogenic and commensal species. They discuss the evolution of AMR, its genetic basis, and possible ways of overcoming it.

The most effective protection from infectious diseases is prophylaxis. While there are several vaccines against enteric infections, such as rotavirus and cholera, development of vaccines against most of them is challenging. In the final article of this Special Focus, Seo *et al*.^3^ discuss recent progress and future challenges in the development of vaccines against gastrointestinal pathogens, both bacterial and viral. Furthermore, they introduce a novel platform for rationally designed enteric vaccines, in which immunogenic epitopes are predicted *in silico* and expressed together in a recombinant vector.

We hope that this Special Focus will add valuable insights into pathogenesis and treatment of gut bacterial infections and suggest directions for further research.
